# Cervicovaginal lipid mediator profiles during high-risk HPV infection associated with subsequent viral clearance or persistence: an exploratory pilot study

**DOI:** 10.3389/fcimb.2026.1954769

**Published:** 2026-10-23

**Authors:** Matteo Mucci, Antonio Recchiuti, Anna-Barbara Moscicki, Domenico Mattoscio

**Affiliations:** 1 Department of Medical, Oral, and Biotechnology Science, University “G. d’Annunzio” Chieti-Pescara, Chieti, Italy; 2 Center for Advanced Studies and Technology (CAST), University “G. d’Annunzio” Chieti-Pescara, Chieti, Italy; 3 Department of Pediatrics, University of California, Los Angeles, Los Angeles, CA, United States

**Keywords:** 12-HETE, 14-HDHA, HPV clearance, HPV persistence, human papillomavirus (HPV), lipid mediators, mucosal immunity, oxylipins

## Abstract

**Background:**

High-risk human papillomavirus (HPV) infection is the primary cause of cervical cancer, whereas most infections are spontaneously cleared. Although local immune and microbial factors associated with HPV outcomes have been investigated, the contribution of lipid mediators within the cervicovaginal microenvironment remains poorly characterized.

**Methods:**

Cervicovaginal wash samples from 30 participants in a prospective longitudinal HPV cohort were analyzed by targeted liquid chromatography–tandem mass spectrometry. Samples were obtained from HPV-negative women, HPV-positive women who were negative for the relevant HPV type at the subsequent visit (subsequent-clearance group), and HPV-positive women in whom the same HPV type remained detectable at the subsequent visit.

**Results:**

Six lipid mediators were detected in cervicovaginal wash samples. During HPV infection, women with subsequent clearance had higher measured concentrations of total 12-HETE and total 14-HDHA than HPV-negative women. In the secondary percentage-based analysis, the relative contribution of PGE_2_ was lower in the subsequent-clearance group than in both the HPV-negative and subsequent-persistence groups, while the relative contribution of total 14-HDHA was higher in the subsequent-persistence group than in HPV-negative women. Direct comparison of correlation coefficients identified an opposite TxB_2_–14-HDHA relationship in subsequent clearance and persistence.

**Conclusions:**

Higher cervicovaginal concentrations of total 12-HETE and total 14-HDHA during HPV infection were associated with clearance of the relevant HPV type at the subsequent visit. The opposite TxB_2_–total 14-HDHA associations observed in subsequent clearance and persistence further suggest outcome-associated differences in lipid-mediator coordination. These exploratory findings identify lipid mediators as candidate correlates of HPV outcomes.

## 1 Introduction

Persistent infection with high-risk human papillomavirus (HPV) is a necessary cause of cervical cancer (Cohen et al., 2019; Tewari, 2025), yet the majority of HPV infections are naturally cleared by the host immune system before progression to clinically significant disease (Moscicki et al., 1998; Gravitt, 2011). While viral persistence is a critical determinant of cervical carcinogenesis, the local mechanisms that promote either viral clearance or persistence remain incompletely understood (Woodman et al., 2007). Previous studies have demonstrated that HPV clearance is associated with changes in cell-mediated immunity and in the cervical microenvironment, including alterations in cytokine production and cervicovaginal microbial communities, suggesting that local mucosal immunity plays a central role in determining infection outcome (Scott et al., 2001; Moscicki et al., 2020). Importantly, recent cervicovaginal metabolic profiling studies further support the concept that there is an interplay between microbial composition, local inflammation, metabolite profiles, HPV infection and cervical disease risk (Ilhan et al., 2019). These studies suggest that complex interactions between HPV, inflammation, and immune responses are hallmarks of HPV interplay with the host and cervical cancer progression. However, little is known about the contribution of lipid mediators to HPV persistence and clearance.

The female lower genital tract represents a complex mucosal ecosystem in which epithelial cells, resident immune cells, microbial communities, and soluble mediators interact to maintain tissue homeostasis and mount antiviral responses (Iwasaki, 2010). Lipid mediators derived from arachidonic acid and omega-3 polyunsaturated fatty acids are potent regulators of inflammation, host defense, and tissue repair, capable of influencing leukocyte recruitment and cytokine production (Bennett and Gilroy, 2016). In addition to initiating inflammatory responses, lipid mediators derived from omega-3 fatty acids actively promote the resolution of inflammation through specialized pro-resolving pathways (Serhan et al., 2015). These mediators facilitate restoration of tissue homeostasis while preserving host defense mechanisms, and defects in pro-resolving responses have been implicated in chronic inflammatory and infectious diseases (Alfaro et al., 2022). Lipids also regulate the activation, differentiation, metabolism, membrane signaling and antigen presentation of immune cells at systemic and local levels, thereby linking lipid production to the regulation of the immune response in health and disease (Garcia et al., 2023).

Among these bioactive molecules, cyclooxygenase (COX)-derived prostanoids and lipoxygenase (LOX)-derived metabolites represent major lipid mediator families that exert complementary and sometimes opposing functions in the regulation of mucosal immunity and tissue homeostasis (Dennis and Norris, 2015). Prostaglandin E_2_ (PGE_2_) and thromboxane B_2_ (TxB_2_) are produced through cyclooxygenase-dependent pathways, whereas leukotriene B_4_ (LTB_4_), 12-hydroxyeicosatetraenoic acid (12-HETE), and 15-hydroxyeicosatetraenoic acid (15-HETE) are generated through lipoxygenase-mediated metabolism of arachidonic acid (Wang et al., 2021). In addition, docosahexaenoic acid (DHA)-derived metabolites such as 14-hydroxydocosahexaenoic acid (14-HDHA) are increasingly recognized as products of DHA metabolism associated with pathways involved in the regulation and resolution of inflammation (Serhan et al., 2009). Together, these lipid mediators constitute interconnected metabolic circuits that coordinate the initiation, amplification, and termination of immune responses. As such, perturbations in the balance of lipid mediator pathways can deeply influence local immune responses and have been linked to ineffective pathogen clearance, chronic inflammation, and disease progression (Ferri et al., 2023; Zhang et al., 2023; D’Orazio and Mattoscio, 2024; Tredicine et al., 2025).

However, whether cervicovaginal lipid-mediator profiles measured during HPV infection are associated with the subsequent course of infection remains largely unknown. We therefore hypothesized that distinct cervicovaginal lipid-mediator signatures are associated with subsequent HPV clearance or persistence, reflecting differences in local inflammatory and pro-resolving immune responses.

To address this question, we performed targeted LC-MS/MS profiling of lipid mediators in cervicovaginal wash samples obtained from women with no detected HPV and from HPV-positive women classified according to type-specific HPV status at the subsequent visit.

## 2 Materials and methods

### 2.1 Study design and study population

This study used archived cervicovaginal wash samples from women enrolled in a prospective longitudinal natural-history study of HPV infection. The study design and population have been detailed previously (Moscicki, 2001; Moscicki et al., 2014). In brief, healthy women aged 13–21 with normal cytology were invited to take part in a natural-history study of HPV, which ran from 1990 to 2014. The women were seen at four- to six-monthly intervals for a sexual behavior interview, collection of a cervicovaginal lavage with normal saline, HPV testing, cytology, and colposcopy. For the present analysis, 30 women were selected and comprised three groups: (i) HPV-negative women (n = 14), with no HPV detected within two years before or after the sampled visit; (ii) subsequent-clearance (n = 6), comprising women who were HPV-positive at the sampled visit and negative for the relevant HPV type at the next visit; and (iii) subsequent-persistence (n = 10), comprising women who were HPV-positive at the sampled visit and positive for the same HPV type at the next visit. Thus, the specimens in both outcome groups were collected during HPV infection, before the subsequent outcome was established. HPV testing was performed based on PCR amplification using Roche Linear Array and an in-house Luminex platform (Moscicki, 2001; Farhat et al., 2015). Cervicovaginal wash specimens were collected by lavage of the cervix and vaginal fornices with sterile saline, recovered, processed according to the parent study protocol, centrifuged to remove cellular debris, aliquoted, and stored at −80 °C until lipidomic analysis. The primary study was approved by the Committee on Human Research, University of California, San Francisco, and participants consented to participate and to place their samples in the repository.

### 2.2 Lipid mediator extraction and LC-MS/MS analysis

LC–MS-grade water, methanol and formic acid were obtained from Sigma-Aldrich/Merck KGaA (Darmstadt, Germany). Cervicovaginal wash samples were analyzed using a targeted lipid mediator metabololipidomics procedure adapted from (Dalli et al., 2018).

Briefly, 1.0-mL aliquots of cervicovaginal wash fluid were spiked with a mixture of deuterium-labelled internal standards to obtain a final internal-standard concentration of 1,000 pg/mL. Samples were mixed with four volumes of ice-cold methanol and maintained at −20 °C for 45 min to promote protein precipitation. Samples were subsequently centrifuged at 2,000 × g for 10 min at 4 °C, and the resulting supernatants were subjected to C18 solid-phase extraction. The methyl-formate fractions containing the lipid mediators were collected, evaporated to dryness under a gentle stream of nitrogen and reconstituted in methanol/water, 1:1, v/v, before LC–MS/MS analysis.

Chromatographic separation was performed using an Agilent 1290 Infinity II UHPLC system coupled to an Agilent 6495D triple-quadrupole mass spectrometer equipped with an Agilent Jet Stream electrospray ionization source. Lipid mediators were separated on a Kinetex PS C18 column (100 × 3.0 mm, 2.6 µm particle size, 100 Å pore size; Phenomenex) maintained at 50 °C. Mobile phase A consisted of water containing 0.1% formic acid, whereas mobile phase B consisted of methanol containing 0.1% formic acid. The flow rate was maintained at 0.500 mL/min.

The gradient was initiated at 45% B and maintained for 2.0 min. Mobile phase B was then increased to 80% at 16.5 min and to 98% at 16.6 min. The column was maintained at 98% B until 18.5 min, after which the composition was changed to 10% B at 18.6 min and maintained until 20.9 min. The total chromatographic run time was 21 min, followed by a 4-min post-run period.

The mass spectrometer was operated in negative-ion dynamic multiple reaction monitoring mode. Source parameters were as follows: drying-gas temperature, 240 °C; drying-gas flow, 12 L/min; nebulizer pressure, 55 psi; sheath-gas temperature, 360 °C; sheath-gas flow, 12 L/min; capillary voltage, −4,000 V; and nozzle voltage, −1,500 V. The fragmentor voltage and collision-cell accelerator voltage were set at 166 and 5 V, respectively. Collision energies and MS1/MS2 resolution settings were optimized for the individual analyte transitions.

Lipid mediators were identified by matching their chromatographic retention times and characteristic MRM transition or transitions with those of authentic standards (**Supplementary Table S1**). The compounds monitored included cyclooxygenase-derived mediators, leukotrienes, hydroxylated arachidonic-acid metabolites, DHA-derived pathway markers and specialized pro-resolving mediators. TxB_2_, PGE_2_, LTB_4_, total 12-HETE, total 15-HETE, and total 14-HDHA met the detection and quantification criteria and were retained for downstream analyses. Hydroxylated fatty acids (12-HETE, 15-HETE, 14-HDHA) were quantified without stereochemical discrimination; their concentrations therefore represent the combined abundance of the corresponding R and S stereoisomers.

External calibration standards were prepared over the range of 39–5,000 pg/mL, corresponding to 39–5,000 ppt or 0.039–5.000 ng/mL. Quantification was performed using the analyte-to-deuterated-internal-standard peak-area ratio. Calibration curves were generated by plotting the normalized detector response against the nominal analyte concentration. Chromatographic peak integration and quantitative calculations were performed using Agilent MassHunter Quantitative Analysis software. Representative chromatograms shown in **Supplementary Figure S1** were selected from cervicovaginal wash samples containing detectable concentrations of the respective lipid mediators.

### 2.3 Statistical analysis

Statistical analyses were performed using GraphPad Prism (version 10; GraphPad Software, San Diego, CA, USA) or Python. Data are shown as individual values with median and interquartile range. Measured lavage concentrations (pg/mL) were designated as the primary outcomes. Overall differences among the three groups were assessed separately for each mediator using the Kruskal–Wallis test, followed by Dunn’s pairwise comparisons. Adjusted p values < 0.05 were considered statistically significant. As a secondary exploratory analysis, each mediator was divided by the sum of the six consistently detected mediators in the same sample and expressed as a percentage. These percentages describe relative representation within the detected six-mediator pool.

Spearman correlation coefficients were calculated for all mediator pairs and displayed as correlation matrices, with yellow boxes indicating nominal within-group associations at p < 0.05. Differences in Spearman coefficients between groups were evaluated directly using two-sided label-permutation tests. Exact enumeration was used for the HPV-negative versus subsequent-clearance comparison (38,760 allocations) and the subsequent-clearance versus subsequent-persistence comparison (8,008 allocations), whereas 100,000 random permutations were used for the HPV-negative versus subsequent-persistence comparison. Benjamini–Hochberg correction was applied separately across the 15 mediator-pair comparisons within each group contrast.

As secondary exploratory analyses, associations of lipid-mediator concentrations with smoking and hormonal contraceptive use were evaluated using rank-based label-permutation tests. To reduce confounding by clinical outcome, labels were permuted within the HPV-negative, subsequent-clearance, and subsequent-persistence groups. Benjamini–Hochberg correction was applied across the six lipid mediators for each clinical variable. HPV genotype was treated as a categorical variable.

p values < 0.05 were considered statistically significant. * p < 0.05; ** p < 0.01; *** p < 0.001.

## 3 Results

### 3.1 Measured lipid mediator concentrations during HPV infection are associated with subsequent outcome

A targeted LC-MS/MS platform was used to quantify lipid mediators in cervicovaginal wash samples. TxB_2_, PGE_2_, LTB_4_, total 12-HETE, total 15-HETE, and total 14-HDHA were detected and quantified (**Supplementary Table S2**). Measured concentrations were compared among HPV-negative women, HPV-positive women with subsequent clearance, and HPV-positive women with subsequent type-specific persistence (**Figure 1**). Women with subsequent clearance had significantly higher measured concentrations of total 12-HETE and total 14-HDHA than HPV-negative women.

**Figure 1:**
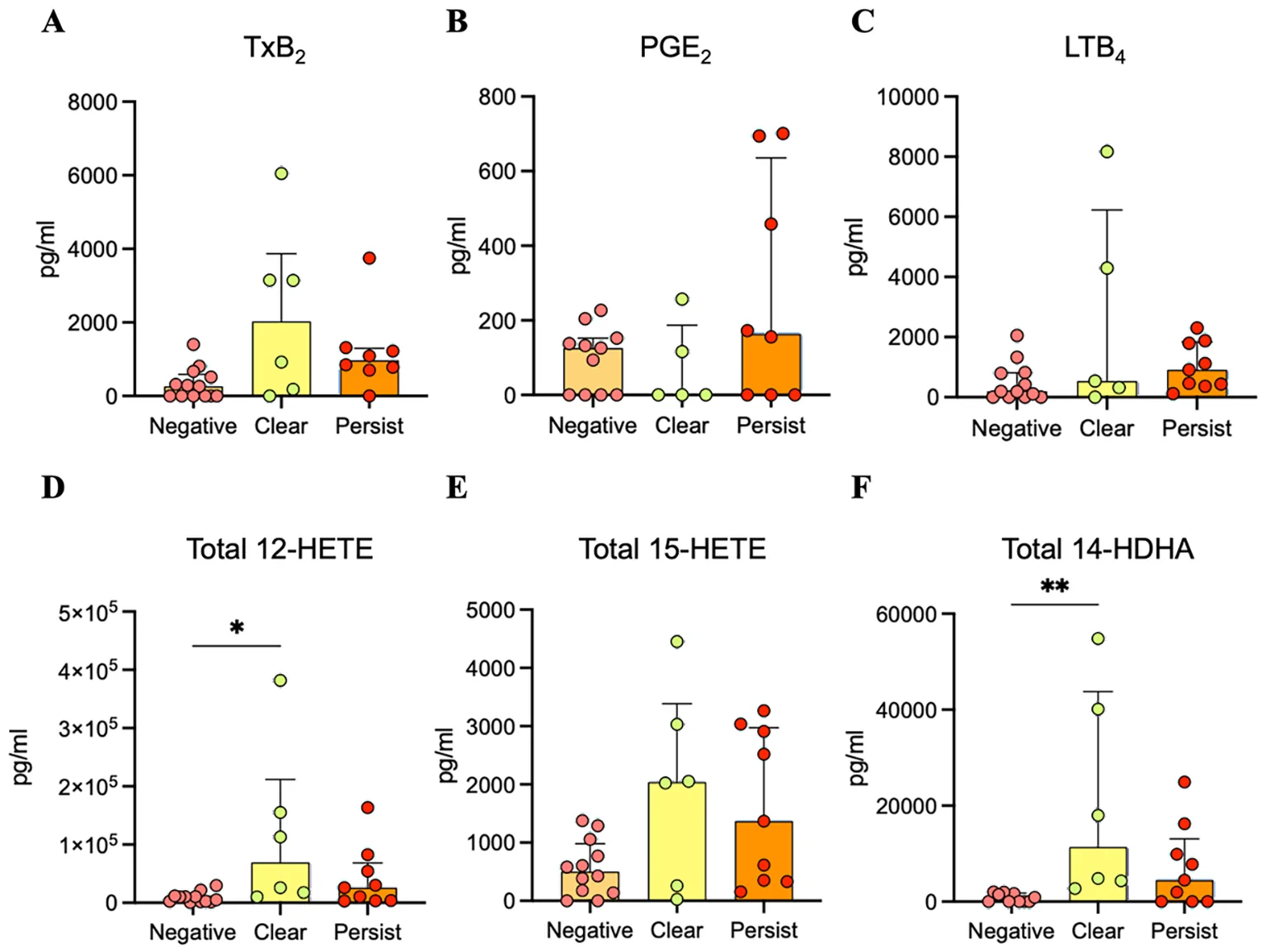
Measured lipid mediator concentrations according to subsequent HPV outcome. **(A-F)** Concentrations of TxB_2_, PGE_2_, LTB_2_, total 12-HETE, total 15-HETE, and total 14-HDHA were measured by targeted LC-MS/MS in cervicovaginal washes from HPV-negative women (negative), HPV-positive women with clearance of the relevant type at the subsequent visit (clear), and HPV-positive women with persistence of the same type at the subsequent visit (persist). Data are presented as individual values, median, and interquartile range. Statistical significance was assessed using the Kruskal–Wallis test followed by Dunn’s *post hoc* multiple-comparison test.

Measured concentrations in the subsequent-persistence group largely overlapped those in the HPV-negative group. Since the clearance-group specimens were collected during the HPV-positive visit, collectively these results indicate a selective enriched production of total 12-HETE and total 14-HDHA in women that subsequently clear the high-risk HPV infection.

To examine whether the observed lipid-mediator profiles might be influenced by available clinical and lifestyle factors, we next assessed their associations with smoking, hormonal contraceptive use, and HPV genotype. Smoking information was available for 25 participants and hormonal contraceptive-use information for 19 participants (**Supplementary Table S3**). No association between smoking and any lipid mediator remained significant after FDR correction (**Supplementary Table S4**). Hormonal contraceptive use was nominally associated with higher TxB_2_ concentrations (median, 1,225.4 versus 464.9 pg/mL; permutation p = 0.0249), but this association did not remain significant after correction across the six mediators (q = 0.149). Genotype-specific analyses were limited by the small number of participants carrying each genotype. In an exploratory comparison of HPV-16/18 versus other detected genotypes, no lipid-mediator difference was significant after FDR correction (**Supplementary Table S3**).

### 3.2 Secondary exploratory analysis of relative representation within the detected mediator pool

To explore the balance among the mediators detected by this targeted assay, each mediator was expressed as a percentage of the sum of the six consistently detected mediators in the same sample (**Figure 2**). The relative contribution of PGE_2_ was lower in the subsequent-clearance group than in both the HPV-negative and subsequent-persistence groups. In contrast, the relative contribution of total 14-HDHA was higher in the subsequent-persistence group than in HPV-negative women. No significant percentage-based differences were observed for TxB_2_, LTB_4_, total 12-HETE, or total 15-HETE.

**Figure 2:**
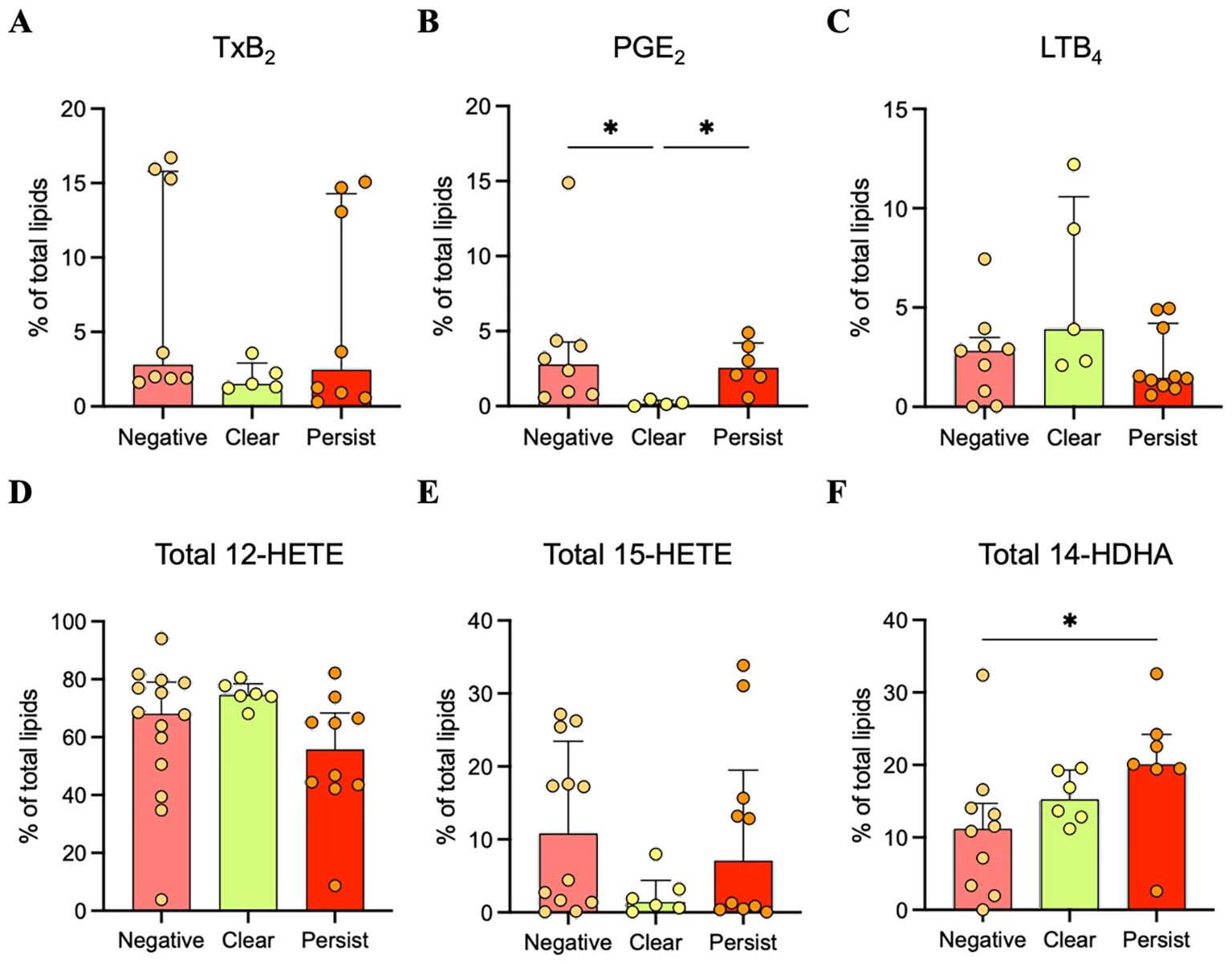
Secondary exploratory analysis of relative mediator representation according to subsequent HPV outcome. **(A-F)** Each mediator was expressed as a percentage of the sum of TxB_2_, PGE_2_, LTB_4_, total 12-HETE, total 15-HETE, and total 14-HDHA detected in the same sample. Data are presented as individual values, median, and interquartile range. Statistical analyses were performed using the Kruskal–Wallis test followed by Dunn’s *post hoc* multiple-comparison test.

Interestingly, the differences observed for total 12-HETE and total 14-HDHA in the absolute concentration analysis were markedly attenuated after normalization to the total detected lipid mediator pool. Similarly, the lower relative contribution of PGE_2_ in the subsequent-clearance group and the higher relative contribution of total 14-HDHA in the subsequent-persistence group suggests that these alterations may occur within the context of a broader shift in lipid mediator production according to the HPV status.

Collectively, these observations suggest that successful HPV clearance may be associated with enhanced lipid mediator biosynthesis at the mucosal level, with a preferential production of total 12-HETE and total 14-HDHA, and by an overall reorganization of the lipid metabolic pathways.

### 3.3 Lipid mediator associations differ according to subsequent HPV outcome

Since lipid mediators are generated through interconnected enzymatic pathways and frequently act in a coordinated manner, we next evaluated pairwise associations among absolute lipid-mediator concentrations within each study group (**Figure 3**).

**Figure 3:**
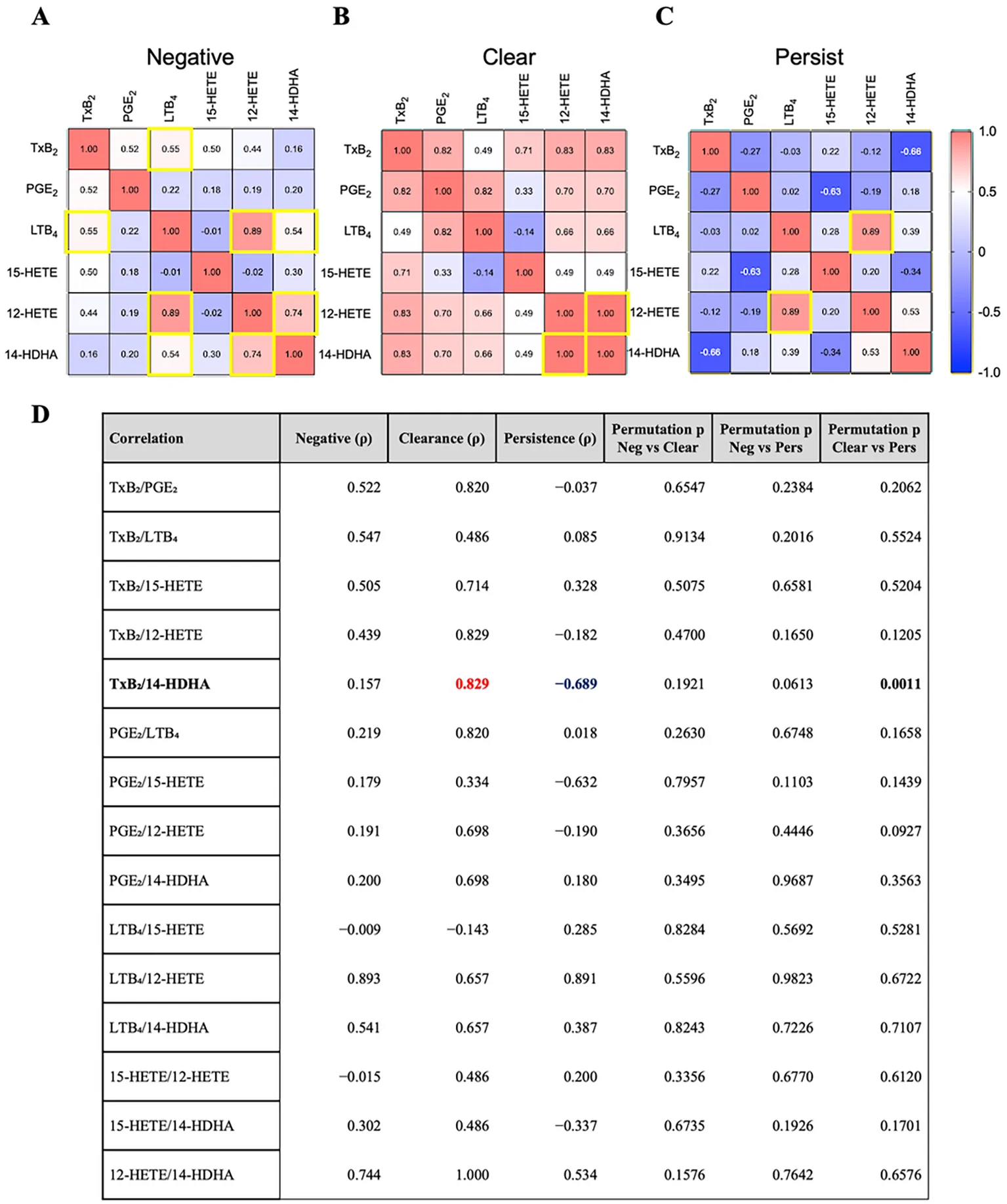
Correlation analysis of measured lipid mediator concentrations according to subsequent HPV outcome. Heatmaps show Spearman correlation matrices in HPV-negative women **(A)**, HPV-positive women with subsequent clearance **(B)**, and HPV-positive women with subsequent type-specific persistence **(C)**. Correlation coefficients are indicated within each cell and color-coded by strength and direction. Yellow boxes identify correlations with FDR < 0.05. **(D)** Spearman coefficients were compared directly between groups using two-sided label-permutation tests. Exact enumeration was used for negative versus clearance and clearance versus persistence, and 100,000 random permutations were used for negative versus persistence.

In HPV-negative women (**Figure 3A**), LTB_4_ correlated positively with total 12-HETE (ρ = 0.893), and total 12-HETE correlated positively with total 14-HDHA (ρ = 0.744). In women with subsequent clearance (**Figure 3B**), total 12-HETE and total 14-HDHA were perfectly rank-correlated (ρ = 1.000). In women with subsequent persistence (**Figure 3C**), the positive LTB_4_–total 12-HETE correlation (ρ = 0.891) is statistically significant. Other nominal within-group associations shown in the heatmaps did not remain significant across the 15 mediator pairs.

To assess these differences, correlation coefficients were evaluated by permutation testing. Direct between-group comparison identified one significant differential correlation (**Figure 3D**). TxB_2_ and total 14-HDHA were positively associated in women with subsequent clearance (ρ = 0.829) but inversely associated in women with subsequent persistence (ρ = −0.689). The between-group difference was significant by exact two-sided permutation testing (Δρ = 1.518, p = 0.0011).

No correlation difference between HPV-negative women and either HPV-positive outcome group remained significant after FDR correction. Thus, this analysis supports a specific difference in the TxB_2_–total 14-HDHA relationship between subsequent clearance and persistence.

Overall, these results provide exploratory evidence that the coordination of selected cervicovaginal lipid mediators measured during HPV infection differs according to subsequent type-specific outcome.

## 4 Discussion

In this pilot study, cervicovaginal lipid mediators were measured at an HPV-positive visit and related to HPV status at the subsequent visit. The principal finding was that women who subsequently cleared the relevant HPV type had higher measured concentrations of total 12-HETE and total 14-HDHA than women with no detected HPV during the two years before or after sampling. The correlation analysis additionally identified an opposite TxB_2_–total 14-HDHA relationship in subsequent clearance and persistence.

12-HETE is produced through arachidonic acid metabolism and has been linked to leukocyte activation, cell migration, and inflammatory regulation (Porro et al., 2014), whereas 14-HDHA is a DHA-derived pathway marker associated with regulation and resolution of inflammation (Wang et al., 2016). The concurrent elevation of these mediators in women who subsequently cleared HPV infection may therefore reflect coordinated activation of both arachidonic acid-derived inflammatory pathways and DHA-derived pro-resolving pathways. Such a pattern is consistent with a balanced mucosal immune response, characterized by sufficient inflammatory activation to promote antiviral defense together with engagement of resolution mechanisms that may prevent the establishment of chronic inflammation and favor viral clearance.

In contrast, women with subsequent HPV persistence did not exhibit comparable concentration changes, suggesting a less pronounced local lipid mediator response during infection. The secondary percentage-based analysis instead showed a higher relative contribution of total 14-HDHA in subsequent persistence than in HPV-negative women, while PGE_2_ contributed a smaller fraction of the detected mediator pool in subsequent clearance than in either comparison group. This broader framework is important because alterations in cervical cytokines and chemokines have previously been linked to HPV infection prevalence (Olukomogbon et al., 2024). Indeed, persistent HPV infection was characterized primarily by alterations in the relative composition of the detected lipid mediator pool according to subsequent HPV outcome, with a lower relative contribution of PGE_2_ in the subsequent-clearance group and a higher relative contribution of total 14-HDHA in the subsequent-persistence group.

In addition, correlation matrices identified a marked divergence in the TxB_2_–total 14-HDHA relationship, namely positive in women with subsequent clearance and negative in women with subsequent persistence. Together, these findings identify candidate lipid mediator features associated with HPV infection outcome (**Figure 4**) and support further investigation of lipid pathways in the regulation of local antiviral immunity.

**Figure 4:**
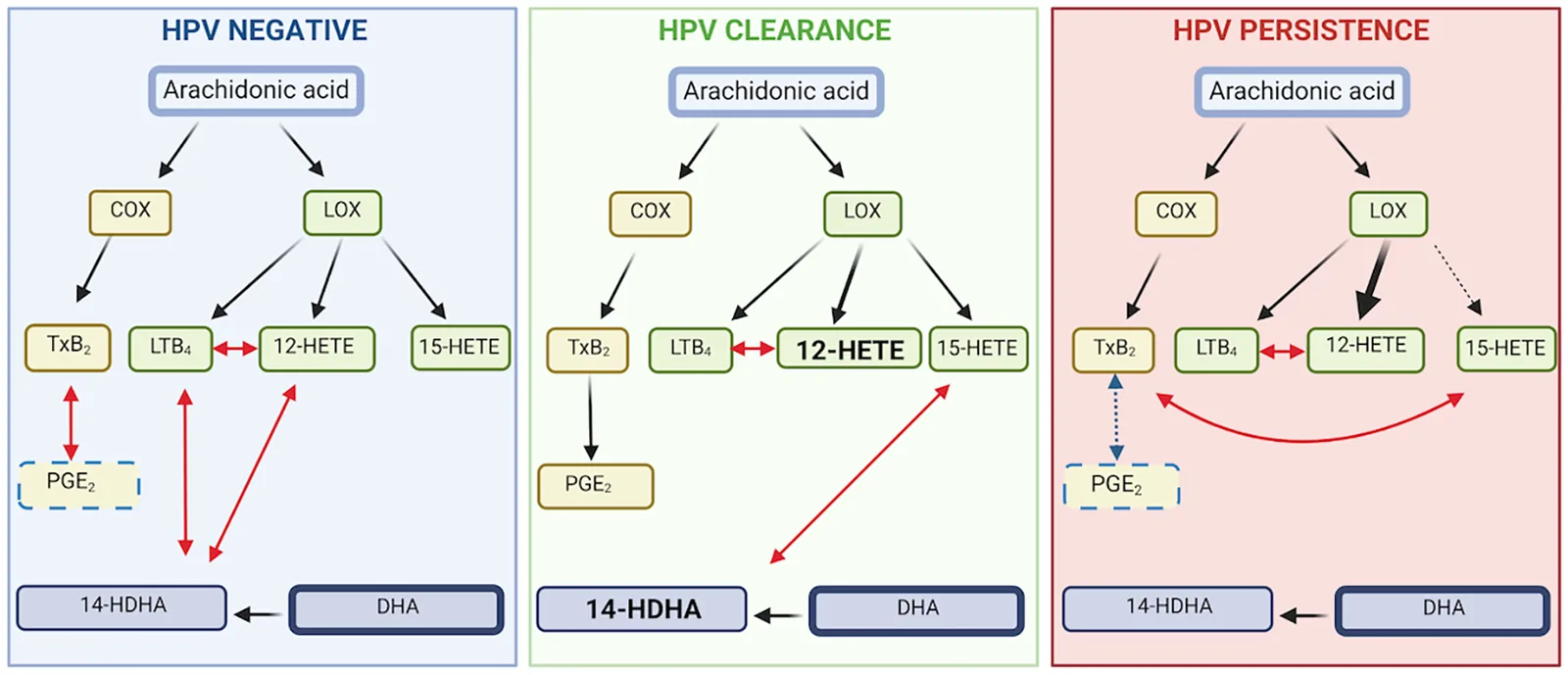
Cervicovaginal lipid-mediator features measured during HPV infection according to subsequent outcome. Bold labels denote total 12-HETE and total 14-HDHA, whose measured concentrations were higher in women with subsequent clearance than in HPV-negative women. Dashed boxes denote secondary percentage-based reduction within the restricted six-mediator pool. The bidirectional connection between TxB_2_ and total 14-HDHA represents the differential correlation observed between subsequent clearance and persistence by exact permutation testing. Created in https://BioRender.com .

This observation may be relevant in the cervicovaginal environment, where immune responses are shaped by interactions among epithelial and immune cells, soluble mediators, and the resident microbiota. Lipid mediators can modulate inflammatory and immune responses (Gilroy and Bishop‐Bailey, 2019), while microbiota composition has been associated with distinct cytokine profiles and differential HPV clearance or persistence (Suárez-Rico et al., 2026). Together, these bidirectional interactions may contribute to the local microenvironment that favors either viral clearance or persistent infection. Overall, these findings support the idea that local mediator profiles may be associated with subsequent HPV outcome.

This study is limited by its small sample size, particularly the subsequent-clearance group, single sample per participant, restricted detected mediator panel, and quantification of hydroxylated fatty acids without stereochemical discrimination. The absence of paired samples immediately before and after the outcome, together with the lack of virological, immunological, microbiological, and clinical covariates, prevents mechanistic interpretation.

In conclusion, higher cervicovaginal total 12-HETE and total 14-HDHA concentrations during HPV infection were associated with clearance of the relevant HPV type at the subsequent visit, whereas subsequent persistence was not accompanied by comparable concentration changes. Thus, successful HPV clearance may be associated with coordinated activation of inflammation-related and pro-resolving lipid pathways, whereas persistence may reflect an inadequate or dysregulated local lipid mediator response. From a translational perspective, the present results identify candidate lipid mediator signatures for future validation. Higher total 12-HETE and total 14-HDHA during infection were associated with subsequent clearance, whereas the relative representation of PGE_2_ and total 12-HETE, together with altered mediator correlations, may identify features associated with subsequent HPV outcome. These findings should be validated in larger longitudinal studies with paired sampling and integrated measures of viral burden, mucosal immunity, and microbiota composition.

## Data Availability

The raw data supporting the conclusions of this article will be made available by the authors, without undue reservation.
