# Supplementary material for: Cervicovaginal lipid mediator profiles during high-risk HPV infection associated with subsequent viral clearance or persistence: an exploratory pilot study
Source: Front Cell Infect Microbiol. 2026 Oct 23;16:1954769. doi: 10.3389/fcimb.2026.1954769 (PMC13739483; doi:10.3389/fcimb.2026.1954769)
Supplement: Supplementary file 1 [file DataSheet1.pdf]

Supplementary Material

Supplementary Tables

Lipid mediator	Precursor ion *m/z*	Quantifier product ion *m/z*	Qualifier product ion *m/z*	Retention time (min)	Collision energy, quantifier/qualifier (V)	Polarity
TxB_2	369.2	195.1	169.0	7.89	12 / 16	Negative
PGE_2	351.2	315.3	271.2	8.35	12 / 16	Negative
LTB_4	335.5	317.0	195.1	12.48	16 / 16	Negative
(±)12-HETE	319.2	179.2	—	15.96	12	Negative
(±)15-HETE	319.2	219.2	—	15.62	12	Negative
(±)14-HDHA	343.2	281.4	205.2	16.04	12 / 12	Negative

Supplementary Table S1. LC-MS/MS parameters used for targeted lipid mediator profiling. Multiple reaction monitoring (MRM) transitions and optimized mass spectrometric parameters used for the LC-MS/MS analysis of selected lipid mediators. The table reports the precursor ion (m/z), quantifier and qualifier product ions (m/z), retention time, collision energies, and ionization polarity of each analyte. TxB_2, PGE_2, and LTB_4 were monitored using both quantifier and qualifier transitions, whereas (±)12-HETE and (±)15-HETE were quantified using a single transition. All analytes were detected in negative electrospray ionization mode.

Lipid mediator	Biosynthetic pathway	% of detection	Interpretation
TxB_2	COX	70	Consistently detected; included in downstream analyses
PGD_2	COX	16.6	Sporadically detected; excluded from downstream analyses
PGE_2	COX	66.6	Consistently detected; included in downstream analyses
LTB_4	5-LOX	83.3	Consistently detected; included in downstream analyses
(±)12-HETE	12-LOX	100	Consistently detected; included in downstream analyses
(±)15-HETE	15-LOX	93.3	Consistently detected; included in downstream analyses
(±)4-HDHA	DHA-derived	23.3	Sporadically detected; excluded from downstream analyses
(±)14-HDHA	DHA-derived	73.3	Consistently detected; included in downstream analyses
(±)17-HDHA	DHA-derived	13.3	Sporadically detected; excluded from downstream analyses
Resolvin D1	SPM	0	Not detected
Resolvin D2	SPM	0	Not detected
Resolvin D5	SPM	0	Not detected
Resolvin E1	SPM	0	Not detected
Lipoxin B_4	SPM	0	Not detected
Protectin D1	SPM	0	Not detected
Maresin 1	SPM	0	Not detected
Maresin 2	SPM	0	Not detected

Supplementary Table S2. Detection frequency of lipid mediators quantified by targeted LC-MS/MS in cervical-vaginal wash samples. Detection frequency was calculated as the percentage of samples with concentrations above the lower limit of quantification (LLOQ) within study population. TxB_2, PGE_2, LTB_4, (±)12-HETE, (±)15-HETE, and (±)14-HDHA were detected and were included in downstream analyses. PGD_2, (±)17-HDHA, and (±)4-HDHA were detected only in a limited number of samples and were therefore excluded from subsequent analyses. Specialized pro-resolving mediators (SPMs), including resolvins, lipoxins, protectins, and maresins, were below the limit of quantification in all study groups.

**Characteristic**	**Overall**	**HPV-negative**	**Clearance**	**Persistence**
Smoking status, n (%)				
Current smoker	10/25 (40.0%)	5/13 (38.5%)	2/5 (40.0%)	3/7 (42.9%)
Non-smoker	15/25 (60.0%)	8/13 (61.5%)	3/5 (60.0%)	4/7 (57.1%)
Missing	5	1	1	3
Hormonal contraceptive use				
Yes	9/19 (47.4%)	3/7 (42.9%)	2/3 (66.7%)	4/9 (44.4%)
No	10/19 (52.6%)	4/7 (57.1%)	1/3 (33.3%)	5/9 (55.6%)
Missing	11	7	3	1
HPV genotype, n		Not applicable		
HPV-16	3	—	0	3
HPV-18	3	—	0	3
HPV-31	2	—	1	1
HPV-45	1	—	0	1
HPV-51	2	—	0	2
HPV-52	1	—	1	0
HPV-56	1	—	1	0
HPV-59	1	—	1	0
HPV-68	2	—	2	0

Supplementary Table S3. Available clinical and lifestyle characteristics according to subsequent HPV outcome. Data are presented as n (%) among participants with available information. Percentages exclude participants with missing data. HPV genotype is reported only for women who were HPV-positive at the sampled visit and is presented according to subsequent type-specific clearance or persistence.

**Comparison**	**Lipid**	**Median group 1 (pg/mL)**	**Median group 2 (pg/mL)**	**Permutation p**
Smoker vs non-smoker (y/n)	TxB_2	685,4	316,0	0,6335
Smoker vs non-smoker (y/n)	PGE_2	416,0	152,3	0,4796
Smoker vs non-smoker (y/n)	LTB_4	1.562,7	539,6	0,2385
Smoker vs non-smoker (y/n)	Total 12-HETE	26.037,3	10.634,0	0,1654
Smoker vs non-smoker (y/n)	Total 15-HETE	611,5	1.055,3	0,5799
Smoker vs non-smoker (y/n)	Total 14-HDHA	5.225,4	1.970,3	0,0751
**Hormonal contraceptive (y/n)**	**TxB_2**	**1.225,4**	**464,9**	**0,0249**
Hormonal contraceptive (y/n)	PGE_2	257,1	135,1	0,0865
Hormonal contraceptive (y/n)	LTB4	916,6	1.224,0	0,9483
Hormonal contraceptive (y/n)	Total 12-HETE	10.766,2	40.165,2	0,4503
Hormonal contraceptive (y/n)	Total 15-HETE	1.368,4	611,5	0,6472
Hormonal contraceptive (y/n)	Total 14-HDHA	2.712,6	3.145,6	0,9658
HPV-16/18 vs other detected genotypes	TxB_2	1.036,5	1.006,5	1,0000
HPV-16/18 vs other detected genotypes	PGE_2	576,4	136,0	0,2822

HPV-16/18 vs other detected genotypes	LTB_4	1.018,7	1.210,9	0,8749
HPV-16/18 vs other detected genotypes	Total 12-HETE	28.053,7	54.320,7	0,9578
HPV-16/18 vs other detected genotypes	Total 15-HETE	1.630,7	2.039,0	1,0000
HPV-16/18 vs other detected genotypes	Total 14-HDHA	6.112,6	11.385,3	0,3111

Supplementary Table S4. Associations of clinical factors and HPV genotype with lipid-mediator concentrations. Associations with smoking and hormonal contraceptive use were evaluated using two-sided rank-based label-permutation tests stratified by clinical outcome (HPV-negative, subsequent clearance, or subsequent persistence). HPV-genotype analysis used an exact two-sided permutation test comparing HPV-16/18 with the other detected genotypes. Nominal p values and FDR-adjusted q values are reported.

Supplementary Figures

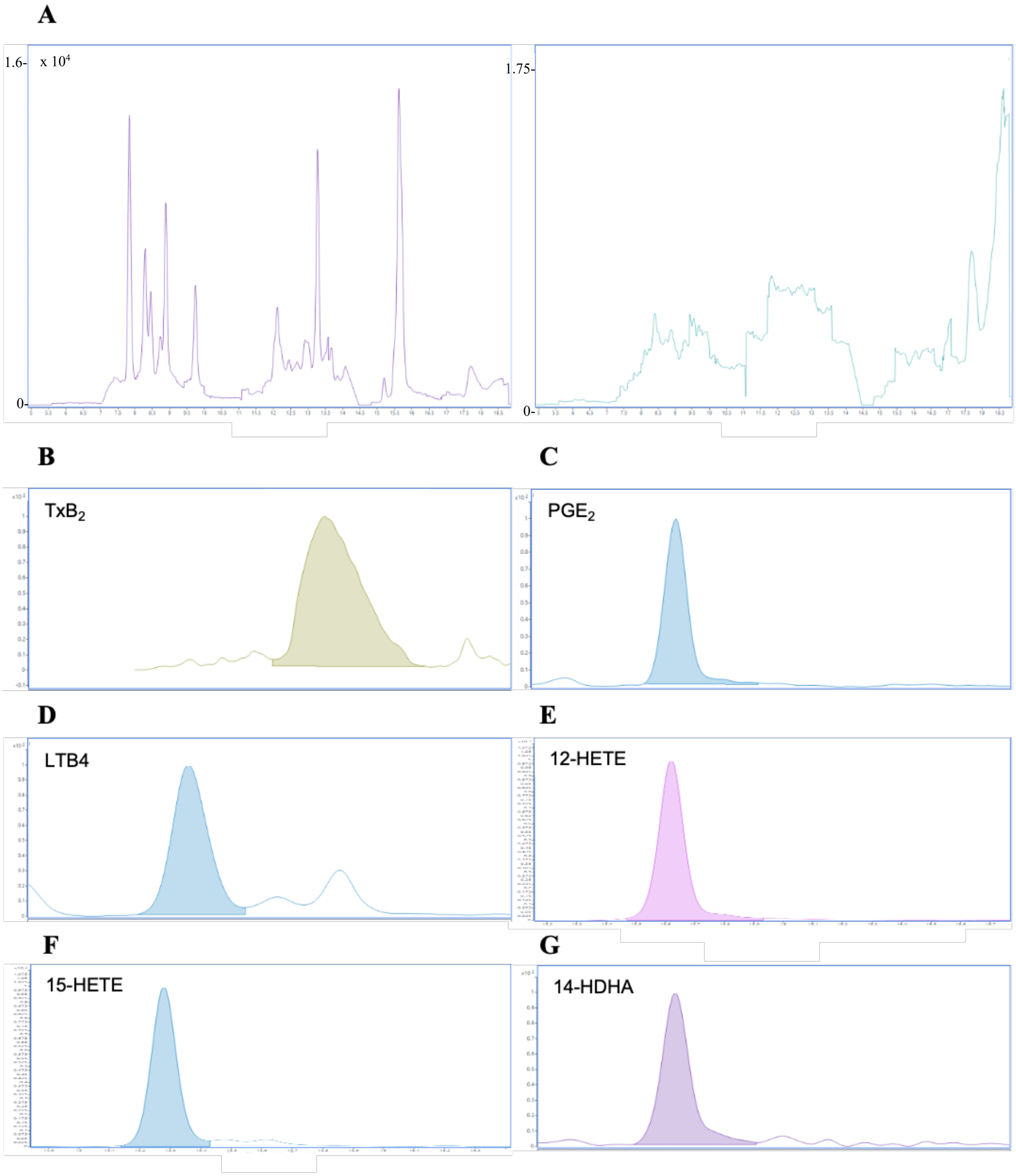

Supplementary Figure S1. Representative MRM chromatograms of lipid mediators detected in cervicovaginal washes samples. A) Representative total ion chromatograms (TICs) acquired in multiple reaction monitoring (MRM) mode from a cervical-vaginal wash sample (left panel) and a methanol blank analyzed under identical chromatographic conditions (right panel). The cervical-vaginal wash sample exhibited multiple well-defined chromatographic peaks corresponding to endogenous lipid mediators included in the targeted analytical platform, whereas the solvent blank showed no comparable analyte-specific signals at the retention times of the detected compounds. **B-**

G) Representative extracted-ion chromatograms obtained by targeted LC–MS/MS analysis of cervicovaginal wash samples for **A)**, TxB_2 (*m/z* 369.2 → 169.0 and 369.2 → 195.1); **B)** PGE_2 (*m/z* 351.2 → 271.2 and 351.2 → 315.3); **C**) LTB_4 (*m/z* 335.5 → 195.1 and 335.5 → 317.0); **D)** ±)12-HETE (*m/z* 319.2 → 179.2; retention time); **E)** (±)15-HETE (*m/z* 319.2 → 219.3); **F)** (±)14-HDHA (*m/z* 343.2 → 205.2 and 343.2 → 281.4). Analytes were detected in negative electrospray ionization mode using dynamic multiple reaction monitoring. Compound assignments were based on agreement of retention times with authentic standards and the presence and co-elution of analyte-specific MRM transitions, when two transitions were available. Traces are representative of samples with concentrations above the analytical detection threshold and are displayed using independently normalized intensity scales (y axes) vs acquisition time (x axes).
